# Bayesian inference based modelling for gene transcriptional dynamics by integrating multiple source of knowledge

**DOI:** 10.1186/1752-0509-6-S1-S3

**Published:** 2012-07-16

**Authors:** Shu-Qiang Wang, Han-Xiong Li

**Affiliations:** 1 Department of Systems Engineering and Engineering Management, City University of Hong Kong, Hong Kong; 2 School of Mechanical and Electrical Engineering, Central South University, Changsha 410083, China

## Abstract

**Background:**

A key challenge in the post genome era is to identify genome-wide transcriptional regulatory networks, which specify the interactions between transcription factors and their target genes. Numerous methods have been developed for reconstructing gene regulatory networks from expression data. However, most of them are based on coarse grained qualitative models, and cannot provide a quantitative view of regulatory systems.

**Results:**

A binding affinity based regulatory model is proposed to quantify the transcriptional regulatory network. Multiple quantities, including binding affinity and the activity level of transcription factor (TF) are incorporated into a general learning model. The sequence features of the promoter and the possible occupancy of nucleosomes are exploited to estimate the binding probability of regulators. Comparing with the previous models that only employ microarray data, the proposed model can bridge the gap between the relative background frequency of the observed nucleotide and the gene's transcription rate.

**Conclusions:**

We testify the proposed approach on two real-world microarray datasets. Experimental results show that the proposed model can effectively identify the parameters and the activity level of TF. Moreover, the kinetic parameters introduced in the proposed model can reveal more biological sense than previous models can do.

## Background

A challenge facing molecular biology is to develop quantitative, predictive models of gene regulation. The advance of high-throughput microarray technique makes it possible to measure the expression profiles of thousands of genes, and genome-wide microarray datasets are collected, providing a way to reveal the complex regulatory mechanism among cells. There are two broad classes of gene regulatory interactions: one based on the 'physical interaction' that aim at identifying relationships among transcription factors and their target genes (gene-to-sequence interaction) and another based on the 'influence interaction' that try to relate the expression of a gene to the expression of the other genes in the cell (gene-to-gene interaction).

In recent years, researchers have proposed many different computational approaches to reconstruct gene regulatory networks from high-throughput data, e.g. see reviews by Bansal et al. and Markowetz and Spang [1,2]. These approaches fall roughly into two categories: qualitative and quantitative aspects. Inferring qualitative regulatory networks from microarray data has been well studied, and a number of effective approaches have been developed [3-10]. However, these methods are based on coarse grained qualitative models [11,12], and cannot provide a realistic and quantitative view of regulatory systems. On the other hand, quantitative modelling for gene regulatory network is in its infancy. Research on quantitative models for genetic regulation has arisen only in recent years, and most of them are based on classical statistical techniques. Liebermeister et al. [13] proposed a linear model for cell cycle-related gene expression in yeast based on independent component analysis. Holter et al. [14] employ singular value decomposition to uncover the fundamental patterns underlying gene expression profiles. Pournara et al. [15] and Yu et al. [16] proposed the Factor analysis model to describe a larger number of observed variables. However, these approaches are based on linear regression, and are not always being consistent with the observations in biochemical experiments which are nonlinear. Imoto et al. [17] proposed a nonlinear model with heterogeneous error variances. This model matches the microarray data well but it is not satisfying enough in revealing more biological sense. Segal et al. [18] proposed a transcription control network based model and apply their model to the segmentation gene network of Drosophila melanogaster. They reveal that positional information is encoded in the regulatory sequence and input factor distribution. However, there is still a little bit of dilemma in the model: the activity level of transcription factors is difficult to be measured or to be identified. Actually, assaying the transcription factors' activity state in a dynamic fashion is a major obstacle to the wider application of the kinetic modeling. TFs' activity levels are difficult to measure mainly due to two technical limitations: TFs are often present at low intercellular concentrations and the changes in their activity state can occur rapidly due to post-translational modifications.

Based on the above description, this paper aims to describe the transcriptional regulatory network quantitatively. In this work, a Bayesian inference based regulatory model is proposed to quantify the transcriptional dynamics. Multiple quantities, including binding energy, binding affinity and the activity level of transcription factor are incorporated into a general learning model. The sequence features of the promoter and the occupancy of nucleosomes are exploited to derive the binding energy. Compared with the previous models, the proposed model can reveal more biological sense.

## Results

### Case Ι: Circadian patterns in rat liver

Circadian rhythm is a daily time-keeping mechanism fundamental to a wide range of species. The basic molecular mechanism of circadian rhythm has been studied extensively. As a real example to test our approach, we considered the dynamics of the circadian patterns in rat liver. We employ the datasets from Almon et al [19]. This experiment was designed to examine fluctuations in gene expression in liver within the 24 hour circadian cycle in normal animals. Fifty-four male normal Wistar rats were housed in a strictly controlled stress free environment with light: dark cycles of 12 hr: 12 hr. Three animals were sacrificed at each of 18 selected time points within the 24 hour cycle. RNA was prepared from livers for gene arrays. Time point designations reflect time after lights on in hours. For details, please refer to Table S1 in additional file 1.

### Analysis of the predicted activity levels of transcription factors

To test the proposed model on the above dataset, we employ two important transcriptional regulators of which activity levels indicate the variation of heat signals in a subset of gene circadian network, hsf1 and ppara. In total, we selected 7 genes to perform posterior inference of TF activities. To ensure identifiability, we included three genes that are regulated solely by hsf1 (HSP110, HSPA8 and COL4A1), and two genes that are regulated solely by ppara (ACSL1 and HMGCS1). The remaining two genes are jointly regulated by hsf1 and ppara. These genes were chosen since they exhibit the largest variance in the microarray time course, and hence are likely to provide a cleaner representation of the output of the system.

**Figure 1:**
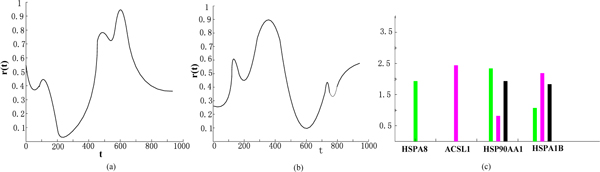
**Results on circadian patterns data**. (a) mean activity profile for hsf1, (b) mean activity profile for ppara, (c) bar-chart representation of the parameters ki, giving the logical structure of the interaction between two TFs.

The inferred TFs' activity levels are shown in Figure 1(a) and 1(b). Both inferred TF profiles show a noisy periodic behaviour [20]. Figure 1(c) gives the values of the parameters k_i _for the four selected circadian genes (HSPA8, ACSL1, HSP90AA1 and HSPA1B). The green column represents the response k_1 _to hsf1 alone, the red column is the response k_2 _to ppara alone and the black column represents the joint response k_12_. It can be seen that, for gene, HSPA8, the model predicts a clear activation by hsfl alone, which is consistent with the experimental conclusion from Yan et al [20]. The black columns of HSP90AA1 and HSPA1B demonstrate that the model predicts a significant combinatorial activation which can be verified by mutagenetic techniques, i.e. by knocking out one of the TFs.

### The biological sense of kinetic parameters

Table 1 shows the relationship between scaling parameter *k *and the corresponding binding affinity *φ*. In table 1, 'H' indicates 'high' and 'L' indicates 'low'. We define the scaling parameter ki as 'High' if it is bigger than the mean value, as 'low', otherwise, and the same to binding affinity *φ*. From Table 1, we can find that, for most case, the scaling parameter is in accordance with the binding affinity: High scaling parameter coupling with high binding affinity, vice versa. However, gene COL4A1 and HSP110 are 2 exceptions: they have high scaling parameter but low binding affinity. Our view is that low binding affinity but high value for *k_i _*might represent a TF which rarely binds to promoter but can strongly regulate gene expression when bound.

**Table 1:** Relationship between scaling parameter k and the corresponding binding affinity φ.

Gene	HSP110	HSPA8	COL4A1	ACSL1	HMGCS1	HSP90AA1-hsf1	HSPA1B- hsf1	HSPA1B- ppara
*k*	H	H	H	H	L	H	L	H
φ	L	H	L	H	L	H	L	H

**Figure 2:**
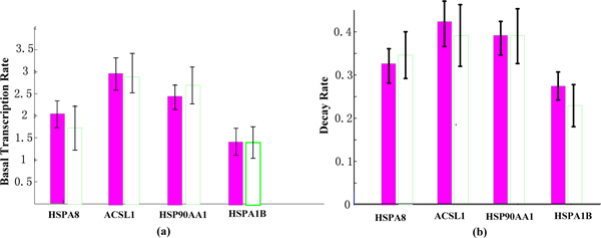
**The bar charts for basal transcription rates and decay rates**. (a) Basal transcription rates from our model and that of Barenco et al. Red are estimates obtained with our model, white are the estimates obtained by Barenco et al [21]. (b) Similar for decay rates.

**Figure 3:**
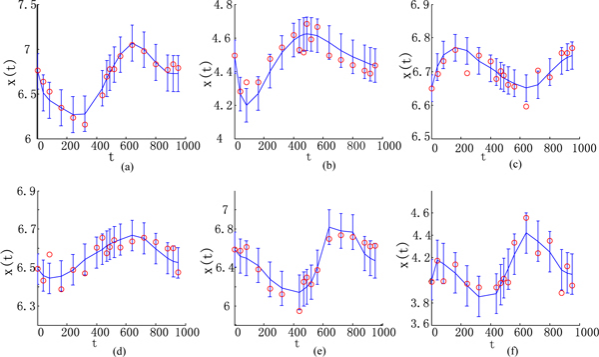
**The predicted mean expression profiles**. (a) HSPA8, (b) COL4A1, (c) ACSL1, (d) HMGCS1, (e) HSP90AA1 and (f) HSPA1B. The red circle indicates the observed value at each time-points.

Figure 2 shows the results of inference on the values of the parameters c_j _and ω_j_. The columns on the left, shaded red, show results from our model and the white columns are the estimates obtained by the method of Barenco et al. [21]. The parameters were assigned a vague gamma prior distribution (a = b = 0.2, corresponding to a mean of 1 and a variance of 5). The results are in good accordance with the results obtained by Barenco et. a l [21]. We can find that the parameters c_j _and ω_j _obtained by our method have smaller variance than that of Barenco et al. Figure 3 shows the fit of the model to the observed data at each time-point.

### Case II: A yeast synthetic network for in vivo assessment

**Figure 4:**
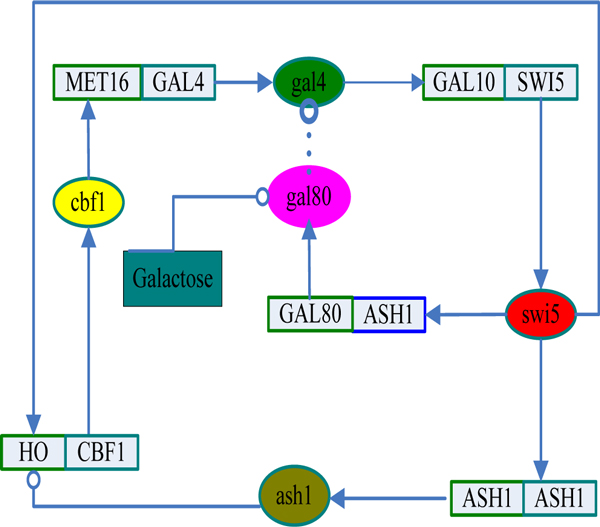
**IRMA network**. The rectangle indicates the gene while the oval represents the protein.

Validation of gene regulation network (GRN) inference methods has traditionally been done using in silico networks. However, depending on how realistic the choice of an in silico model is, this kind of validation approach has obvious limitations. To our knowledge, rarely the underlying model from which artificial/simulated data is generated is realistic enough. Real biological networks are fairly complex chemical reaction network models. In simulation setting one typically adds noise on top of a hypothetical simulation model, but the noise characteristics may not be realistic enough. Also, simulation models tend to be overly simplistic when compared to e.g. real gene regulatory networks. Data measured from a real biological system is, real. To overcome these problems, we use the IRMA network to evaluate out model. The IRMA network is a synthetically constructed GRN in the Saccharomyces cerevisiae genome [22], which is designed to be maximally independent in such a way that genes in the network are not regulated by genes outside of the network (i.e. by other yeast genes). However, genes in the IRMA network may regulate other genes in the genome. The network consists of five genes and there are positive and negative feedback loops and one protein to protein interaction, shown as Figure 4. Although the IRMA network contains only five genes, there are studies suggesting that the performance on smaller networks typically generalize to larger networks [1,23]. The data samples were collected every 20 min up to 5 hr in five independent experiments for the switch-on state, and every 10 min up to 3 hr in four independent experiments for the switch-off state. For details on the construction of the network and experimental procedures, we refer to [22]. One of the purposes of the IRMA network is to provide a realistic benchmark set for computational studies by providing mRNA-level measurements from a known GRN. To our knowledge, the IRMA network and dataset are the first of a kind that are meant for validation purposes. Besides, we assume that mRNA decay rates may be gene-specific, but remain constant in time [36]. The sequences of all promoters are retrieved from SCPD and SGD database. The scanning region ranges from -800 to +50 bp of each target gene.

### Analysis of the predicted activity levels of transcription factors

**Figure 5:**
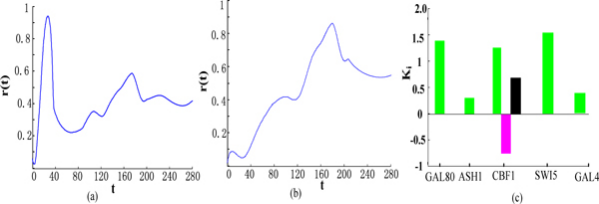
**Results on IRMA network data**. (a) mean activity profile for regulator swi5, (b) mean activity profile for regulator ash1, (c) bar-chart representation of the parameters ki.

To evaluate whether the proposed model can effectively learn the TFs' activity level and the regulation type, we first evaluate the model using the switch-on time series data. The inferred TFs' activity levels are shown in Figure 5(a) and 5(b). Both inferred TF profiles show a noisy switch-on behavior. Figure 5(c) gives the values of the parameters ki for the five target genes. The green column represents the response to the first regulator alone, the red column is the response to the second regulator alone and the black column represents the joint response, k_12_. It can be seen that, for gene, GAL80, the model predicts a clear activation by swi5 which is consistent with the experimental conclusion [22]. For gene CBF1, the red downward column indicates that ash1 behaves as a repressor, which is verified in [22]. The black column of CBF1 demonstrates that the model predicts a significant combinatorial regulation [22].

### Analysis of the kinetic parameters

Table 2 shows the relationship between scaling parameter k and the corresponding binding affinity φ. In table 2, the definition of 'High' and 'Low' are same as in Table 1, and the same abbreviations are employed. It can be found that gene GAL80 has the TF that rarely binds to promoter but can strongly up-regulate its expression when bound.

**Table 2:** Relationship between k and φ for IRMA network data.

Gene	GAL80	GAL4	SWI5	ASH1	CBF1-swi5	CBF1-ash1
k	H	L	H	L	H	H
φ	L	L	H	L	H	H

**Figure 6:**
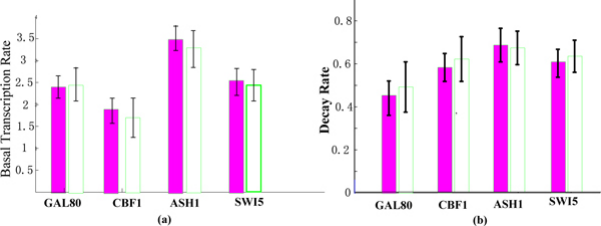
**The bar charts for basal transcription rates and decay rates**. (a) Basal transcription rates from our model and that of Opper et al. [24]. Red are estimates obtained with our model, white are the estimates obtained by Opper et al. (b) Similar for decay rates.

Figure 6 shows the results of inference on the values of the parameters c_j _and ω_j_. The columns on the left, shaded red, show results from our model and the white columns are the estimates obtained by Opper et al. [24]. It can be found that the results are in good accordance with the results obtained by the method of Opper et Al. [24]. It can be found that the parameters c_j _and ω_j _obtained by our method have smaller variance than that of Opper et al. [24].

**Figure 7:**
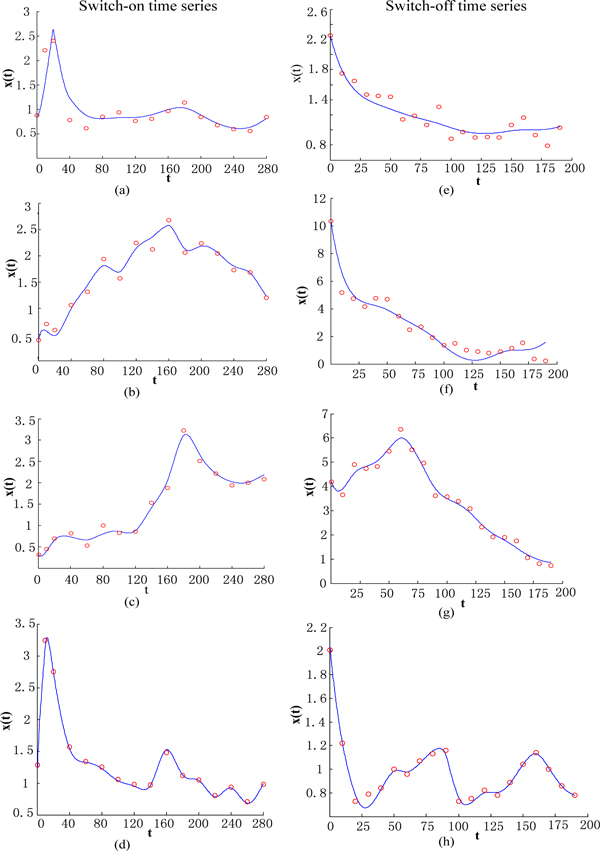
**The predicated mean expression profiles**. Expression profile and mean reconstruction of target genes. Switch-on time series: (a) GAL80, (b) ASH1, (c) CBF1, (d) GAL4, (e)-(h) The same genes in switch-off time series. The red circle indicates the observed value at each time-point.

For comparison, we also evaluate the model using the switch-off data. Figure 7 shows the fit of the model to the observed data at each time-point for both the switch-on case and switch-off case.

## Discussion

In this study, two real-world microarray datasets were exploited two evaluate the efficiency of the proposed model. Comparison shows that the kinetic parameters obtained by our method have smaller variance than that of Barenco et al. [21]. One reason is that the proposed model provides a principled method for the incorporation of prior biological knowledge. This may be in the form of suitable ranges for kinetic parameters, known kinetic parameter values and suitable distributions on measurement noise. Besides, it is possible for the proposed model to circumvent the need for expensive sampling-based inference and a TFA profile can be inferred over all time, rather than just at the discrete time-points at which expression was measured.

The Bayesian inference based model of transcription rates and regulator activity levels allows us to handle these biologically relevant quantities despite the indirect measurement of the former and the lack of measurements of the latter. It also allows us to handle the inherently noisy measurement in a principled way. However, the proposed model still abstracts away some of the explicit processes that generate the actual observed expression data. A more explicit modelling of these will provide a more principled treatment of different sources of noise in the data. Furthermore, this model does not handle directly the upstream events that affect regulator activity. In fact, the current model is an open loop system, such that the regulation of regulator activity is itself viewed as exogenous to the system. By developing a richer modeling language we may capture more complex reaction models, model the upstream regulation of activity levels, and identify systems that involve feedback mechanisms and signalling networks.

Post-Transcriptional Modification Model (PTMM) have been previously used to model TF activities [25]; in that work, further dependencies were included between TF mRNA expression levels and their predicted activities, which enabled to predict possible post-transcriptional modifications in TFs. Naturally, it should be possible to combine both our approach and their approach to give a model capable of simultaneously inferring TF activities, combinatorial interactions and post transcriptional regulations.

## Conclusions

In this work, we have proposed a computational model to reverse engineer simultaneously both the activity of TFs and the logical structure of promoters by integrating multiple sources of knowledge, including time-series gene expression data, TFs' binding information and sequence features of promoters. The approach relies on a detailed model of transcription, which is an approximation to the Michaelis-Menten model from classical enzyme kinetics, and therefore should be able to capture accurately the effects that changes in TF activity have on gene expression dynamics. We testify the proposed approach on two real-world microarray datasets. Experimental results show that the proposed model can effectively identify the parameters and the activity level of TF. Moreover, the kinetic parameters introduced in the proposed model can reveal more biological sense than previous models can do.

## Methods

### Problem statement

**Figure 8:**
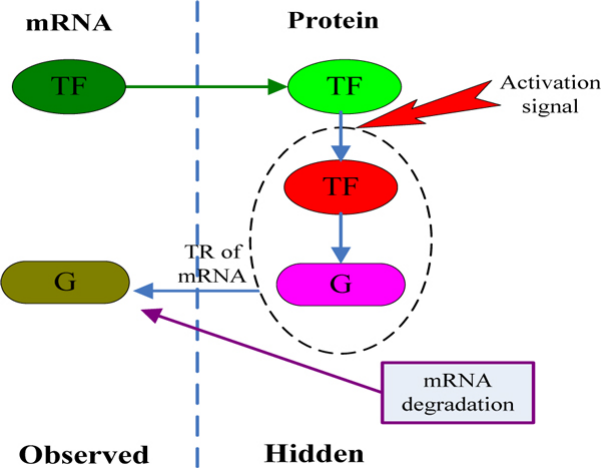
**A qualitative molecular model of transcriptional regulation**. mRNA encoding a transcription factor (TF) is translated to protein. The protein is activated and induces the transcription of a target gene at a certain rate (G). The final accumulation of G mRNA levels is determined by this transcription rate and by the rate of G's mRNA degradation.

A microarray experiment only measures the "observed" quantities, as shown in Figure 8, whereas the other quantities are not observed ("hidden"). The dashed oval encloses the closest quantities on the path between the TF and the target gene. Consider a transcriptional network of n genes that are regulated by m regulators, as well as a time-dependent external signal. Given the structure G and a set X of transcription rates of these genes in T time points, is it possible to reconstruct the time-varying activity level of m regulators, R, at all time points and the corresponding model parameters? This is an infinite dimensional problem that we tackle by placing a stochastic process prior over the activities of regulators.

**Figure 9:**
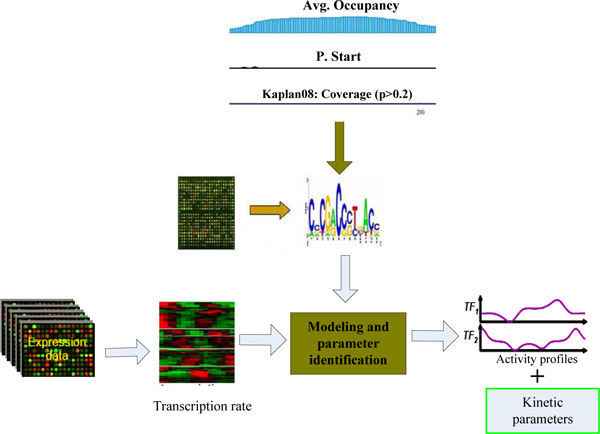
**The framework of proposed method**. The expression data and sequence features of promoters are incorporated into a general learning model. The outputs of the model are kinetic parameters and the activity levels of transcription parameters.

Our approach relies on a continuous time, differential equation description of transcriptional dynamics where TFs are treated as latent on/off variables and are modelled using a switching stochastic process. The framework of the proposed method is shown in the Figure 9.

### Kinematic model of regulation

Compared with the gene expression level, the gene transcription rate can capture more dynamic characteristics of transcription regulation. We here employ the transcription rate to model the regulation function. We first assume:

• The derived transcription rates are average rates over a cell population.

• The speed of a TF's binding to or dissociation from its target sites is assumed to be much more rapid than the transcription process, thus rapid-equilibrium approximation can be used.

Based on the above assumptions, the transcription rate of a gene is proportional to the amount of the gene bound by its regulators in all genes of the measured cell population. We first consider the case that a gene is regulated by a single activator. The corresponding regulation function can be properly described by Michaelis-Menten equation:

(1) $$\frac{dx}{dt}=\beta \frac{r\left(t\right)}{d+r\left(t\right)}+c-\omega x,$$

here x represents the mRNA concentration for a particular gene, *r(t) *the concentration of active TF, *β *and *d *are kinetic constants, *c *a baseline expression rate and *ω *the mRNA decay rate.

To incorporate the sequence feature and the TF binding preference into the model, we set the binding affinity *φ *= k/d, and (1) can be reformulated as

(2) $$\frac{dx}{dt}=\beta \frac{k\varphi r\left(t\right)}{1+k\varphi r\left(t\right)}+c-\omega x,$$

here *k *is a scaling parameter.

We now take the regulation involving two regulators into account. Denote by r_1_(t) and r_2_(t) the concentration of two regulators, *φ*_1 _and *φ*_2 _the binding affinity of two regulators from their own target sites, the regulation function can be written as below:

(3) $$\frac{dx}{dt}=\beta \frac{k_{1}\varphi_{1}r{\left(t\right)}_{1}+k_{2}\varphi_{2}r_{2}\left(t\right)+k_{3}\varphi_{1}\varphi_{2}r_{1}\left(t\right)r_{2}\left(t\right)}{\left(1+\varphi_{1}r_{1}\left(t\right)\right)\left(1+\varphi_{2}r_{2}\left(t\right)\right)}+c-\omega x,$$

Considering the general case, a gene is regulated by n regulators. There are 2^n ^different binding states in total. The n-dimension binary vector is employed to indicate a certain binding state, e.g., a 4-dimension vector (0 1 0 1) indicates that the second and the fourth regulators are bound to their own target sites while the first and the third are not bound. The regulation function can be written as:

(4) $$\frac{dx_{j}}{dt}=\beta_{j}\frac{\sum_{s\in S_{j}}k_{s}\prod_{s_{i}=1;i=1,n}\varphi_{ij}r_{i}\left(t\right)}{\prod_{i=1}^{n}\left(1+\varphi_{ij}r_{i}\left(t\right)\right)}+c_{j}-\omega_{j}x_{j}$$

where S_j _denotes the set of all 2^n ^possible state vectors, and s_i _is the i_th _element of the state vector *s*.

### Modelling for binding affinity

Measuring affinities of molecular interactions in high-throughput format remains problematic, especially for transient and low-affinity interactions. We here try to describe the landscape of binding affinity in the perspective of binding energy between the various DNA-binding molecules and their target genes. Binding affinity landscapes describe how each molecule translates an input DNA sequence into a binding potential that is specific to that molecule. The presented framework models several important aspects of the binding process.

By allowing molecules to bind anywhere along the input sequence, the entire range of affinities is considered, thereby allowing contributions from both strong and weak binding sites [26,27].

• Conventional cooperative binding interactions can be explicitly modelled by assigning higher statistical weights to configurations in which two molecules are bound in close proximity.

• The cooperativity that arises between factors when both nucleosomes and transcription factors are integrated is captured automatically [28].

We first consider the simplest case that there is only one target site S_ij _for TF *i *in the promoter of gene *j*:

$$TF_{i}+S_{ij}\underset{k_{d}}{\overset{k_{b}}{↔}}\left[TF_{i}\cdot S_{ij}\right]$$

The site-specific binding affinity is given by

(5) $$\varphi =C_{i}e^{-\frac{E_{ij}}{kT}}$$

where C_i _is a constant, E_ij _the binding free energy between TF_i _and the promoter of gene *j, k *and *T *are the Boltzmann constant and temperature, respectively.

**Figure 10:**
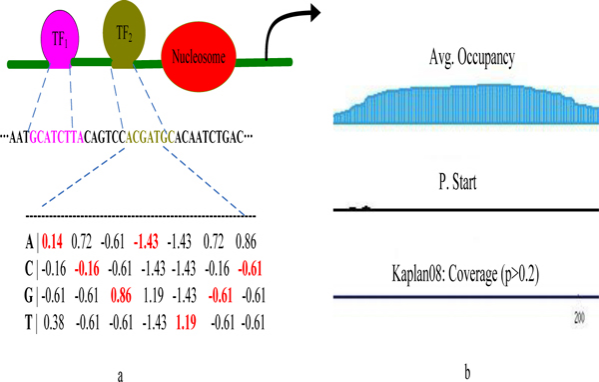
**Employing sequence features and the occupancy of nuclesomes to estimate the binding affinity**. The positional weight matrices are used to represent the sequence motif. The binding may occur anywhere along the input sequence, the entire range of affinities is considered.

The above case can be expanded to the general case that binding may happen in anywhere along the input sequence and the accessibility of target sites varies due to the occupancy of nucleosomes. The general binding affinity is modelled as

(6) $$\varphi_{ij}=C_{i}\overset{N}{\underset{n=1}{\sum }}{p_{ij}}^{\left(n\right)}e^{\frac{-{E_{ij}}^{\left(n\right)}}{kT}}$$

where *p^(n)^_ij _*is the probability of transcription factor *i *binding to the nth binding site in the promoter of gene *j*. Note that the derivation of *p^(n)^_ij _*involves the information of the possible occupancy of nucleosomes. The nucleosomes positions can be predicted based on the nucleosome-DNA interaction model proposed by Kaplan et al [29]. Figure 10(b) shows the occupancy of nucleosomes for the genomic sequence shown in the Figure 10(a).

Since the positional weight matrices (PWM) are often used to represent the sequence motif [30,31], we estimate the binding energy in perspective of PWM. As the background information has been taken into the PWM, the binding free energy can be approximately calculated as below:

$${E_{ij}}^{\left(q\right)}=K^{\left(q\right)}\overset{L}{\underset{l=1}{\sum }}\underset{n=\left\{\left(A,C,G,T\right\}\right)}{\sum }{J^{n}}_{l}\left({M_{L}}^{*}-M_{nl}\right)$$

where ${J^{n}}_{l}=\left\{\begin{array}{c} 1\;if\;n=s\left(l\right)\\ 0\text{ otherwise} \end{array}\right)$

here K^(q) ^is the scaling factor, M*_L _indicates the maximum background frequency in the motif, *s(l) *is the nucleotide in position *l*.

### Regulatory network modelling using dynamic Bayesian inference

In many biological processes, the transcription factor transit from inactive to active state quickly as a consequence of fast post-translational modifications, (the time scale is micro second), so it is reasonable that we model the TF activity as a binary variable r(t) ∈{0,1}[24,32].

For the regulation involving a single regulator, the TF activity can be seen as a two states Markov Jump Process. Based on Ref [24,33], the probability of the system being in a particular state at a given time is given by the following Master equation:

(7) $$\frac{dp_{1}\left(t\right)}{dt}=n_{+}p_{0}\left(t\right)-n_{-}p_{1}\left(t\right)$$

(8) $$\frac{dp_{0}\left(t\right)}{dt}=n_{+}p_{1}\left(t\right)-n_{-}p_{0}\left(t\right)$$

here p_1_(t) = p(r(t) = 1), p_0_(t) = p(r(t) = 0) and *n*_± _indicates the transition rate.

**Figure 11:**
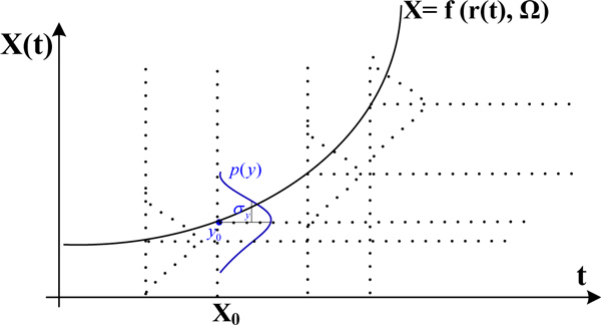
**Normally distributed observational data**. The solid line indicates the mean predicted expression while the dotted line represents the normally distributed observations.

The observed expression data is often assumed to be normally distributed [24]. We now define a noise model that relates the predicted mRNA concentration to the observed expression data, as shown in Figure 11.

Setting y_j_(t) as the observations of mRNA species *j *at time *t*, x_j_(t) the predicted expression and σ_j _the variation, the noise model can be described as

$$y_{j}\left(t\right)\left|r\left(t\right)\sim \right)N\left(x_{j}\left(t\right),\;{\sigma^{2}}_{\text{j}}\right)$$

Based on Refs [24,33], we define the TF's switching stochastic process as the prior distribution, then we combine the prior distribution and the noise model (likelihood) into Bayes' theorem to obtain the posterior over the process:

$$p\left(r\left|y,\;\Omega \right)\right)=\frac{1}{S}p\left(y\left|r,\right)\;\Omega \right)p\left(r\right)$$

where *y *denotes collectively the observations, *Ω *are the parameters involved in the regulation function and *S *a normalization constant.

### Variational inference and model optimization

We will use a variational formulation of the inference problem [34]. Variational inference is a powerful inference method and it has been well applied for optimization by Opper and Sanguinetti [24,33]. Our model optimization is based on Ref [22]. Variational inference is used as an approximation technique: given an intractable probability distribution *p*, the variational approach finds an optimal approximation *q *within a certain family of distributions. This is usually done by minimizing the Kullback-Leibler (KL) divergence between the two distributions

(9) $$KL\left[q∥p)\right]=E_{q}\left[\text{log}\frac{q}{p}\right]=\int \text{log}\frac{q\left(r\right)}{p\left(r\right)}q\left(r\right)dr$$

By selecting a suitable family of approximating distributions, the inference problem is then turned into an optimization problem. It can be shown that the KL divergence is a convex functional of *q *and is equal to zero iff q = p [24,35]. In this case, we will choose the approximating process *q *to be again a Markov Jump Process, so that the required *KL *is given by

(10) $$KL\left[q{∥p)}_{post}\right]=KL\left[q{∥p)}_{prior}\right]+\text{log}S-E_{q}\left[\text{log}p\left(y\left|r,\;\Omega \right)\right)\right]$$

here *S *is a normalization constant, *E_q_*[log *p*(*y*|*r*, Ω)] the expectation of the likelihood of the observations under the approximating process.

According to Ref [24], minimization of the KL functional (11) can be represented as the saddle point problem

(11) $$J=\underset{\tau }{\text{max }}\underset{q}{\text{min}}\left\{KL\left[q∥p_{prior})\right]+\overset{n}{\underset{j=1}{\sum }}\left[\tau_{j}\left(y_{j}-\bar{x}\left(t_{j}\right)\right)-\frac{\sigma^{2}}{2}{\tau_{j}}^{2}\right]\right\}$$

here *τ *is auxiliary variables. It can be found that this functional is concave in *τ *and convex in q. Hence we can exchange min and max. Performing the max first yields the result. This also shows that there is only a unique saddle point solution.

The optimization procedure is based on a forward-backward procedure, leading to ordinary differential equations which can iteratively be solved. Taking the regulation involving two regulators for example, the free energy is a functional of both the approximating processes q^1^, q^2 ^and their transition rates n_1_, n_2_. However, these are not independent, but are related by the Master equation. To incorporate this constraint, we add Lagrange multipliers as

(12) $$L\left(q^{1},q^{2},g_{1},g_{2}\right)=J\left[q^{1},q^{2},g_{1},g_{2}\right]+\int_{0}^{T}\left[\frac{d{q_{1}}^{1}\left(t\right)}{dt}+\left(n_{1-}+n_{1+}\right){q_{1}}^{1}\left(t\right)-n_{1+}\right]\lambda_{1}\left(t\right)dt+\int_{0}^{T}\left[\frac{d{q_{2}}^{1}\left(t\right)}{dt}+\left(n_{2-}+n_{2+}\right){q_{2}}^{1}\left(t\right)-n_{2+}\right]\lambda_{2}\left(t\right)dt$$

where g_1 _and g_2 _are the rates of jumps from the 0 to the 1 state for process q^1 ^and q^2^, respectively.

**Figure 12:**
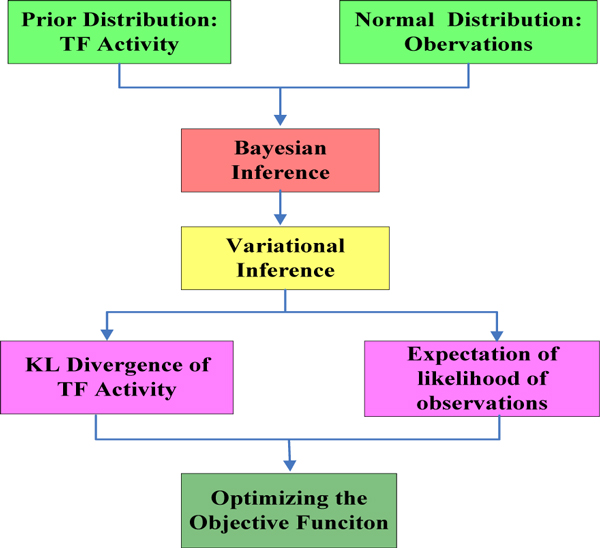
**The framework of the inference**.

The Lagrange multiplier functions obey the final condition λ(T) = 0. Estimation of the parameters *A *and *b *can be done directly by maximizing the approximate marginal likelihood E_q_[*logp(y|r,Ω*)]. The framework of the inference is shown in the Figure 12.

## Competing interests

The authors declare that they have no competing interests.

## Authors' contributions

SQW proposed the method, performed the analysis; HXL supervised the work and revised the paper critically for important intellectual content.

## Supplementary Material

Additional file 1 **Table S1**. The time series gene expression data for circadian patterns in rat liver. Click here for file
